# The Victorians were still faster than us. Commentary: Factors influencing the latency of simple reaction time

**DOI:** 10.3389/fnhum.2015.00452

**Published:** 2015-08-20

**Authors:** Michael A. Woodley of Menie, Jan te Nijenhuis, Raegan Murphy

**Affiliations:** ^1^Department of Psychology, Technische Universität ChemnitzChemnitz, Germany; ^2^Center Leo Apostel for Interdisciplinary Studies, Vrije Universiteit BrusselBrussels, Belgium; ^3^Work and Organizational Psychology, University of AmsterdamAmsterdam, Netherlands; ^4^School of Applied Psychology, University College CorkCork, Republic of Ireland

**Keywords:** secular trend, simple reaction time, methods variance, Galton, processing speed

Woods et al. (2015) claim that secular Simple Reaction Time (SRT) slowing (Woodley et al., 2013), disappears once modern studies are corrected for software and hardware lag, and once Galton's data are corrected for fastest-response selection. Here, this is challenged with a reanalysis of the secular slowing of SRT in the UK amongst large (*N* > 500), population-representative age-matched (≊18–30 years) studies.

Starting with Galton's sample, this is assigned the simulated value estimated by Dodonova and Dodonov (2013, who like Woods et al. were critical of secular SRT slowing, owing to measurement issues) on the basis that he collected the fastest of three trials (207.5 ms). The two sexes in Galton's study are combined (as in Woods et al.), raising the weighted sample mean to 208.5 ms.

Next is the Wilkinson and Allison (1989) study, which attempted to replicate Galton's study one century later, collecting SRTs as part of an exhibit in the London Science Museum. An electronic chronoscope recorded SRTs on magnetic tape, and sampled over eight trials with micro-processor-determined variable foreperiods. The mean SRT value for the 1189 participants aged between 20 and 29 is 245 ms. The presence of long and variable foreperiods necessitates a penalty of 10 ms (Dodonova and Dodonov, 2013). Another 10 ms should be deducted based on key-pressing time (Dodonova and Dodonov, 2013), reducing the mean to 225 ms.

The studies of Deary and Der (2005) and Der and Deary (2006) are also included. The first utilized the highly representative Scottish Twenty-07 cohort. Dodonova and Dodonov (2013) identified a 53 ms lag stemming from liquid crystal stimulus onset delay. This is subtracted from the weighted average of the two sexes (300.8 ms), along with another 10 ms for key-pressing time. The resultant mean is 237.8 ms.

Dodonova and Dodonov (2013) cleaned the male data in the Der and Deary (2006) study, collected from the representative UK Health and Lifestyle Survey, by removing cases for which SRT standard deviations exceeded those for choice RT. This reduced the *N* from 834 to 661, and also reduced the mean from 300 to 284 ms. The estimate was also penalized for LCD onset delay and key-pressing time, reducing the mean to 221 ms. When the SRT value for the female sample is penalized equivalently the resulting value is 239 ms. In order to simulate the female *N* for the purposes of taking a weighted average of both sexes, the actual female *N* is reduced in proportion to the male *N* (79.3% = 881), yielding a weighted mean of 230.9 ms for a combined sample-size of 1472. Table 1 presents the data used in the analysis.

**Table 1:** **SRT means, sample sizes and sampling years for four large, age-matched UK samples**.

**Study**	**Mean SRT (ms)**	***N***	**Mid-range**
			**(Sampling years)**
Galton (1890)	208.5	3418	1888.5 (1884–1893)
Wilkinson and Allison (1989)	225	1189	1980
Der and Deary (2006)	230.9	1472	1984.5 (1984–1985)
Deary and Der (2005)	237.8	543	1987.5 (1987–1988)

Consistent with Dodonova and Dodonov (2013), *N*-weighted regression is employed, as the only data on sample variability is sample size. Figure 1 illustrates the secular trend in British SRT spanning 100 years.

**Figure 1:**
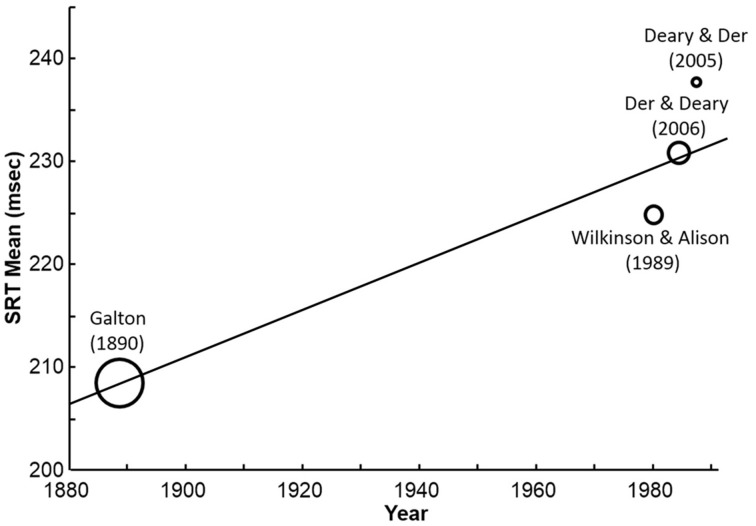
**Secular SRT slowing across four large, representative studies from the UK spanning a century**. Bubble-size is proportional to sample size. Combined *N* = 6622.

The secular slowing between UK studies is statistically significant (β = 0.97; 95% *CI* = 0.969–0.971, *N* = 6622), at +22.8 ms a century.

Additional evidence for generational SRT slowing comes from Verhaeghen (2014), who suggested that the ratio of longitudinal to cross-sectional age-related slowing might indicate generational changes in processing speed. Verhaeghen reports ratios for two SRT studies (0.91 and 1.15), implying both secular losses and gains. For the study of Deary and Der (2005), the SRT ratios are “censored because they were excessively large” (p. 256). In this study, the ratio of the cross-sectional slowing trend (taking the weighted average of all paired between-cohort differences rescaled in terms of change per decade for males and females), to the weighted average decadal longitudinal slowing trend for both the males and females is 0.73, for an *N* of 1926 (*cf*. Woodley et al., 2014, for a detailed reanalysis of this dataset utilizing curve-fitting). The weighted average of the three SRT “Verhaeghen ratios” is 0.9 (*N* = 4078)—tentatively consistent with generational declines (i.e., a ratio of < 1).

In conclusion, Woods et al. (2015) have undoubtedly made an important contribution to the debate concerning the role of software and hardware lag in the inflation of contemporary estimates of SRT, however, the evidence for generational SRT slowing remains quite compelling.

## Conflict of interest statement

The authors declare that the research was conducted in the absence of any commercial or financial relationships that could be construed as a potential conflict of interest.
