# Supplementary material for: The Victorians were still faster than us. Commentary: Factors influencing the latency of simple reaction time
Source: Front Hum Neurosci. 2015 Aug 20;9:452. doi: 10.3389/fnhum.2015.00452 (PMC4542533; doi:10.3389/fnhum.2015.00452)
Supplement: Supplementary file 1 [file Presentation1.PDF]

Online supplement to: The Victorians were still faster than us.

Commentary: Factors influencing the latency of simple reaction time

Michael A. Woodley of Menie[1,2]*

[1]Department of Psychology, Technische Universität Chemnitz, Chemnitz, Germany

[2]Center Leo Apostel for Interdisciplinary Studies, Vrije Universiteit Brussel, Brussels, Belgium

Jan te Nijenhuis[3]

[3]Work and Organizational Psychology, University of Amsterdam, Amsterdam, The Netherlands

Raegan Murphy[4]

[4]School of Applied Psychology, University College Cork, Cork, Republic of Ireland

*michael.woodley-of-menie@hrz.tu-chemnitz.de

Supplement Overview

After a constructive and rigorous round of reviews, the reviewers indicated that they were satisfied with our commentary on the Woods et al. (2015) paper, however it was clear that there were several technical issues raised in the commentary that nonetheless required unpacking. Given the small allotment of space for *Frontiers* commentary articles (1000 words) associate editor Prof. Guillaume A. Rousselet proposed that we provide a more detailed overview of the arguments made in the commentary in the form of an online supplement.

Prior to writing the now published commentary, a somewhat longer version was produced in which the arguments advanced in the commentary were unpacked in considerably greater detail. This earlier version is presented here as a supplement in order to give added context and depth to the published version of the commentary.

Introduction

The publication of Woodley et al. (2013a), in which a meta-regression against time of the means of 16 estimates of simple reaction time (henceforth SRT) yielded evidence of a secular decline of -77ms, spanning 115 years, attracted considerable interest from both within and outside of the scientific community. Several papers were published challenging the finding on various theoretical and methodological grounds (Dodonova & Dodonov, 2013; Flynn, 2013; Nettelbeck, 2014; Parker, 2014; Silverman, 2013). Subsequent defences of the finding were mounted, in which data were reanalysed taking into account the various criticisms, and evidence of secular declines in SRT

were still found to be evident in the data (Woodley et al., 2014a; Woodley of Menie et al., 2015).

The latest critical commentary on Woodley et al. (2013a) comes from Woods and co-workers. A key finding of Woods et al. (2015) is that there exists considerable methods variance (i.e. variance between studies resulting from differences in instrumentation, sampling and data-recording criteria; Jensen, 2006) associated with various forms of computer software and hardware lag, which artificially increase the latencies of modern SRTs. Using two different populations (a community sample of 1469 individuals from New Zealand, aged between 18 and 65, and a sample of 189 individuals from California aged between 18 and 82, recruited in part from the internet), they found that by carefully estimating the various sources of methods variance they were able to reduce the latencies associated with the two estimates considerably – yielding values of 213ms for the New Zealand and 220ms for the US sample (p. 10). Woods et al. (2015) claim that "based on Galton's notebooks, Dordonova [*sic*] and Dordonov [*sic*] (2013) argued that Galton recorded the shortest-latency SRT obtained out of three independent trials per subject." (p. 7). They go on to posit (in line with Dodonova & Dodonov, 2013) that such a data-selection protocol would have led to a substantial overestimation of the capabilities of Galton's 19th century sample. Correction for this is therefore argued to bring Galton's 19th century estimates almost perfectly in line with those reported for the two more recent samples, which would falsify the argument put forward in Silverman (2010) and Woodley et al. (2013a) to the effect that SRT performance has in fact declined appreciably since the 19th century.

Did Galton take three measures per subject and select the minima?

The claim that Dodonova and Dodonov (2013) based their minima of three trial data selection hypothesis on Galton's notebooks is a curious one however, as these researchers appear to have based this instead on two indirect sources of evidence. The first was an article written by Cattell (1890), in which he proposed selecting the minima on a best-of-three basis, and also described how he hopes that the various methodological innovations discussed in the paper would "meet his [Galton's] approval" (p. 373). The second was an article in which Galton described an earlier variant of his mechanical chronoscope as having had the capacity to "make three successive trials with the eye and three with the ear in less than three minutes" (Galton, 1889, p. 306). Dodonova and Dodonov argue that taken together, these sources imply that Galton utilized a three-trial approach coupled with selection for the minima.

There are however legitimate doubts as to whether Galton used selection for the minima, which were addressed in Woodley et al. (2014a) – a follow up to the original Woodley et al. (2013a) paper in which Dodonova and Dodonov (2013) along with three other critical papers were addressed theoretically and empirically. To recap the relevant arguments therein, firstly, Cattell's suggested method is presented as an innovation in methodology – one of many that he hopes Galton will approve. This indicates that Galton was not using this specific data selection method, but some other. Secondly, whilst Galton described an older version of his chronoscope as having had the capacity to take three successive trials, this does not indicate that he actually took three successive trials *per subject* during the period in which he was

collecting data at the Kensington laboratory, nor does it tell us how those three trials were handled in terms of selecting which scores to report, in the event that he actually did this.

Galton's notebooks also indicate that he had read Donders' seminal 1868 paper (see: Galton, 1873), in which he cautions RT experimenters (in translation) that "it would certainly be dangerous to rely only on the minima"; and also: "We have never neglected to determine also the difference in the averages. They protect us from the profound error to which a thoughtless use of the minima could lead" (Donders, 1868/1969, p. 428). Perhaps therefore Galton deliberately implemented his data selection strategy in such a way so as to avoid these "profound errors" associated with "thoughtless use of the minima".

Indeed, there are several alternative ways in which the data from the three trials could have been handled. It is unlikely that the average value was recorded, as Pearson (2014) reports that Galton never utilized averages, therefore this can be ruled out. It is also unlikely that he utilized the median value, as this would have increased the reliability of his SRT measures (Dodonova, pers. comm). This leaves two fairly obvious alternative data selection strategies. Firstly, the first two trials may have been treated as practice or calibration trials (this seems plausible as, given their purely mechanical nature, Galton's chronoscopes may have necessitated constant adjustment and maintenance), and that only the third trial may have been recorded. Alternatively, the intermediary magnitude of the three values may have been taken, on the premise that the lowest and highest values represented flukes or outliers.

It is not possible to know precisely whether or not Galton took three trials from his subjects at the Kensington laboratory, or if he did, precisely how he treated the data. This information is lost to science, as Galton never documented his precise methodology. In the absence of any concrete evidence that would lead us to strongly suspect that Galton was selecting for the minima from among three trials, it seems reasonable therefore to discount this speculation in favour of the more conservative view that Galton recorded one trial per person, based simply on the observation that only one datum was recorded per participant, which is the view of historians of RT such as Johnson et al. (1985) and Jensen (2006).

Given these observations, the conclusion to which Woods et al. (2015) jump concerning the equivalence of their present day samples to those of Galton's era must be considered premature. If Galton did not select the minima, then a mean value of <200ms for the Kensington sample does not seem unreasonable. Consistent with this are the data presented in Johnson et al. (1985), who report RT means for 3460 males aged 18 to 60+, and 1097 females aged 18 to 40. The weighted average of the means reported for the males is 186.88ms, and for the females it is 192.42ms. These values have been corrected upwards for a small calibration error in Galton's conversion chart (3.5ms) described in Dodonova and Dodonov (2013). Therefore the mixed-sex 19th century sample would have had an SRT mean of 190ms (rounding upwards to the nearest centisecond).

This mean would be significantly faster than the values reported by Woods et al. (2015) for either of their samples – even if an exceptionally large standard deviation (*SD*) value is assigned meta-analytically to each sample, such as was done in

Woodley et al. (2013a). In this study, the *SD* value reported by Deary et al. (2001) for their comprehensively representative Scottish sample (119.7ms) was corrected for indirect range restriction on the basis of comparing the Alice Heim 4's *SD*, reported in the same study, with that reported in the manual sample of secondary school students. The larger value of the latter resulted in an upwards correction of the SRT SD to 160.4ms.

Utilizing formulas from Lipsey and Wilson (2000) for calculating the standardized mean difference, the difference (*d*) between Galton's estimates and the estimates from the New Zealand sample equals -0.14 (95% CI = -0.20 to -0.09). The difference between Galton's estimate and the US sample equals -0.19 (95% CI = -0.33 to -0.04). The differences equate to a decline of 22.5ms in 126 years when the New Zealand sample is compared with that of Galton, and 30.5ms for the US sample (i.e. *d**160.4).

What if Galton did take the minima? A reanalysis of secular trends in the UK

Would selecting the minima from only three trials actually make that much of a difference to the finding of a secular trend? Dodonova and Dodonov (2013) employed a sophisticated simulation to determine the effects of this data selection protocol on Galton's sample mean and proposed, based on the results, that it should be adjusted upwards by 21ms. With the correction for the calibration error, this yields a mean of 207.5ms for the males. In considering this, Woodley et al. (2014a) conducted a meta-regression involving six methods-variance-corrected US and UK studies, in which this value was used instead of the 187ms value for Galton's sample. It was found that whilst it attenuated the secular loss in SRT (21ms), the result was nonetheless

significant, suggesting that a secular decline would likely be present, even had Galton selected for the minima of three trials. A subsequently published thresholding analysis revealed that the decline trend remained significant with values for Galton's sample mean as high as 213.85ms (Woodley of Menie et al., 2015).

To further explore the effects of a higher latency value for Galton's study, we look for secular trends amongst a sample of four large, population-representative British studies of SRT. This analysis is intended to build on the previous analyses by demonstrating that the initial finding is essentially robust to changes in the composition of the analysis is in terms of which samples are included, choice of statistical method and correction for methods variance. Furthermore, in previous analyses we eliminated studies based on a criterion of close bio-cultural matching (Woodley et al., 2014a). Here we take this matching one step further by comparing only British studies.

British studies were selected on the basis that they were large (N>500) and strove to be representative of the populations from which they were drawn, and in that respect were comparable to Galton's Kensington sample, which sampled from across seven different social classes, including unskilled workers. Galton himself was agnostic as to how representative of the population as a whole his sample may have been, however he cautioned against taking this objection to unreasonable levels (Galton, 1885). The potential representativeness of Galton's sample can nonetheless be estimated by comparing the height data reported for 20-21-year-old males across social classes (average = 66.93 inches; Johnson et al., 1985, Table 12, pp. 892) with the population-level UK height for the period 1886 to 1890 (coincident with the data

collection period at the Kensington laboratory) calculated for male Army recruits aged 20 (i.e. 66.5 inches; corrected for truncation as reported in Hatton & Bray, 2010, p. 411). This finding strongly suggests that Galton's sample was reasonably representative, especially given that the male standard deviation in height is about 3 inches (the mid-range value reported in Garcia and Quintana-Domeque, 2007; Table 5A, for various European countries, corresponds to a standard deviation of 2.93 inches), which indicates that Galton's sample would have been about 0.14 of a standard deviation taller than the Army sample, a relatively small difference.

We assign to Galton's sample the simulated value estimated by Dodonova and Dodonov (2013) on the basis that he used the minima of three trials, i.e. 207.5ms. Consistent with Woods et al's (2015) approach of combining the sexes in their analyses, on the basis that the sex differences in their comparisons were negligible, we combine the two sexes in Galton's study, which raises the weighted sample mean to 208.5ms. Next, we incorporate a study not considered in our previous meta-analyses, namely that of Wilkinson and Alison (1989), which attempted to replicate Galton's study one century later by setting up an SRT-measuring system as part of a special exhibit on Medicines for Man in the London Science Museum, which ran between the 6th of August and 2nd of November in 1980 (Medicines for Man Organizing Committee, 1980). Wilkinson and Alison note several parallels between the demographics of their own sample and those of Galton's Kensington sample, including age (predominantly 20-30 in both studies) and also large sample size (5,325 participants). Woodley et al., (2013b) have observed other similarities between the demographics of the Wilkinson and Alison sample and the Kensington sample, including comparable socio-economic and ethnic demographics (based on

contemporaneous data on museum visitor and tourism demographics in the UK), and also the presence of a small fee that both sets of subjects would have to have paid in order to participate in the respective exhibits.

Wilkinson and Alison utilized a relatively simple electronic chronoscope (the Unprepared Simple Reaction Time test) which recorded SRT values on magnetic tape, sampled over eight trials with micro-processor-determined variable foreperiods of between 1 and 10 seconds. The display utilized a numerical LED millisecond counter, which began counting upwards after the illumination of a warning signal, and could be arrested by the push of a button. The counter would then display the SRT value for a second or so before extinguishing until the next foreperiod elapsed. The device collected data on SRT and also age of participant, however data on participant sex were not collected. SRT values shorter than 120ms were eliminated as outliers from participant responses. Those with five or more SRT values of longer than 1000ms or one longer than 2500ms were eliminated on the assumption that these participants were not taking the experiment seriously. Also single SRT values that were greater than twice as long as the average value were replaced with the average value. The mean SRT value for the 1,189 participants aged between 20 and 29 was approximately 245ms (as reported in Silverman, 2010). The presence of long and variable foreperiods should, based on Dodonova and Dodonov (2013), necessitate a penalty of 10ms. Another 10ms should be removed from the mean based on key-pressing time (Wilkinson and Alison suggest that key pressing should not add substantially to the SRT values, however simulations conducted by Dodonova and Dodonov suggest that travel distances that are typical of press-buttons, i.e. four or so millimetres, can increase apparent latency by around 10ms). The new value for

Wilkinson and Alison's 1980 mixed-sex Science Museum sample should therefore be 225ms.

The studies of Deary and Der (2005) and Der and Deary (2006) are also included in the analysis. The first is the Scottish Twenty-07 cohort, which sampled across several socio-economic and age classes using a sampling strategy that was designed to achieve maximum representativeness. Dodonova and Dodonov (2013) identified a major source of methods variance in this study in the form of stimulus onset display, which results from a temperature/delay time relationship associated with liquid crystal displays. This onset delay results in a 53ms lag, which must be subtracted from the weighted average of the two sexes (300.8ms), along with another 10ms penalty for key-pressing time. This results in a mixed-sex sample value of 237.8ms for Deary and Der (2005).

Dodonova and Dodonov (2013) subjected the male data from the study of Der and Deary (2006), which was originally collected as part of the UK Health and Lifestyle survey, to a cleaning protocol, removing distorted data for which SRT standard deviations exceeded those for choice RT. This reduced the sample size from 834 to 661 individuals and also resulted in a reduction of the mean from 300ms to 284ms. The mean was also penalized for onset delay, as it utilized an LCD-based device, and key-pressing time, resulting in a mean of 221ms. When the SRT value for the female sample is penalized equivalently this results in a value of 239ms. In order to simulate the female *N* for the purposes of taking a weighted average of both sexes, the actual female *N* is reduced in proportion to the male *N* (79.3%=881). This yields an SRT mean value of 230.9ms for a combined simulated sample-size of 1472. Table 1

presents the data used in this analysis along with their sample sizes and sampling years.

Table 1

SRT means, sample sizes and sampling years for four population representative UK samples

Study	***Mean SRT (ms)***	***N***	***Midrange sampling year (sampling years)***
Galton (1890)	208.5	3418	1888.5 (1884-1893)
Wilkinson & Alison (1989)	225	1189	1980
Der & Deary (2006)	230.9	1472	1984.5 (1984-1985)
Deary & Der (2005)	237.8	543	1987.5 (1987-1988)

Consistent with Dodonova and Dodonov (2013), we reanalyse the secular trend using a fixed effects regression with weighting by *N*. As Dodonova and Dodonov observe, the lack of standard deviation data for all samples means that the only source of information concerning sample variability is sample size. The analysis is implemented using meta-analytic software available on statstodo.com. Figure 1 presents a graph illustrating the secular trend towards slowing SRT in the UK over 100 years.

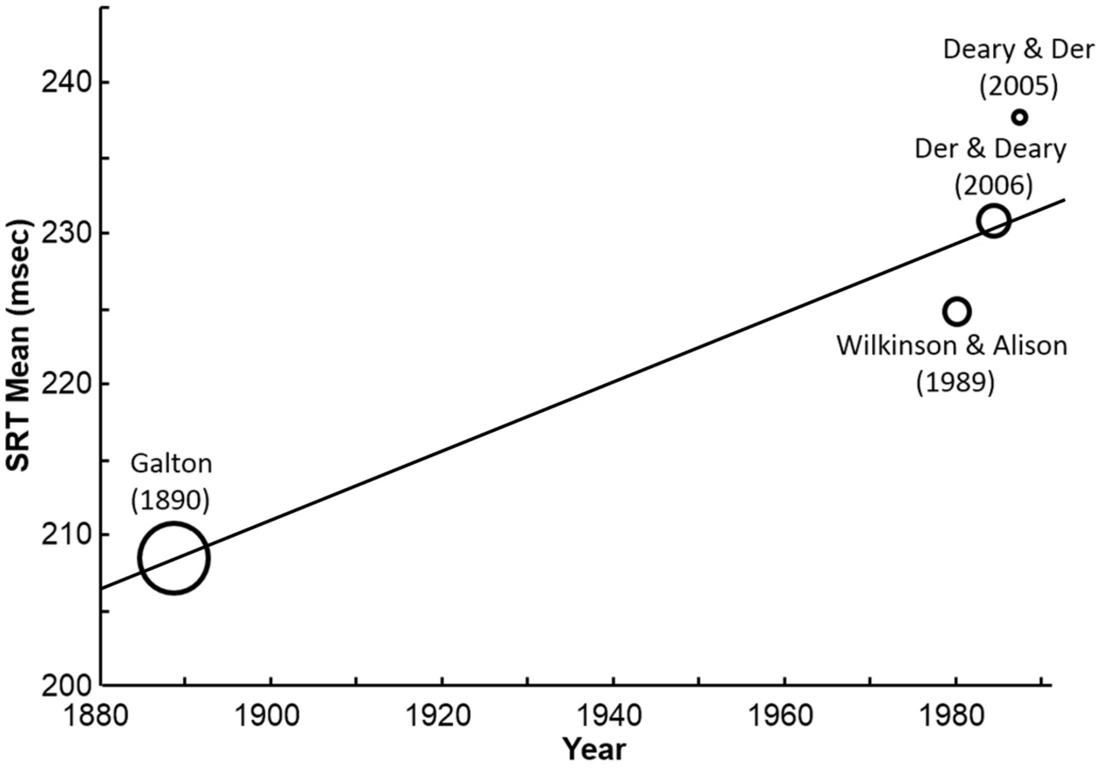

Figure 1. *The secular trend towards slowing SRT across four large, population-representative studies from the UK spanning a century. Bubble-size is proportional to sample size. Combined N=6622.*

The secular slowing between studies is monotonic and statistically significant (β=0.97; 95% CI=0.969 to 0.971, N=6622), and amounts to a latency increase of +22.8ms over 100 years in the UK.

Consistent with the theorized role of genetic selection against general intelligence (*g*) and accumulating mutations in generating this decline in processing speed (Woodley et al., 2013a), this trend can be converted into an equivalent decline in *g* by dividing the decline in ms between the start and approximate end points of the trend (i.e. 1889 and 1989) by the standard deviation in SRT for those aged 18-30. A good reference

estimate for these values comes from the sample aged 24 from Deary and Der (2005), who found values of 78 and 73ms for their male and female sample, respectively, using a balanced sampling strategy designed to maximize representativeness and minimize range restriction. Combining these standard deviation values yields a mixed sex value of 75.3ms. Dodonova and Dodonov (2013) caution against upward correction of standard deviation values from SRT studies based on indirect range restriction, as unequal skew between variables can lead to misleadingly large resultant *SD*s. Consistent with this caution, we do not attempt an indirect correction for range restriction, and simply divide the decline by the uncorrected *SD* value from Deary and Der (2005), which yields a *d* value of 0.3. Removing the correction for indirect range restriction from the disattenuated IQ*SRT correlation reported in Woodley et al. 2013 (the original correlation was reported in Deary et al. 2001) reduces the parameter estimate to .41 (from .54 in Woodley et al., 2013a). Division by this number yields the change in *g* over 100 years, which equals 10.95 points, or 1.1 points per decade. This change falls within the range of values determined on the basis of a meta-analysis of declines in *g* due to genetic selection coupled with the additive effects of mutation accumulation, estimated using the effect of paternal age on offspring *g* as a proxy for *de novo* mutations (i.e. -1.92 to -.53 points per decade; Woodley of Menie, 2015).

Other evidences of slowing SRT: 'Verhaeghen ratios'

An alternative and straightforward method for estimating secular trends in SRT and related processing speed paradigms, involves comparing longitudinal with cross-sectional (i.e. between sample) aging effects. This method was first proposed by

Verhaeghen (2014), who argued that when the cross-sectional decline between cohorts is smaller than the longitudinal decline due to ageing, the longitudinal slowing trend amongst the younger cohorts, when extrapolated out to the age of the older cohorts would indicate that they are relatively slower, hence the presence of secular declines can be inferred, and vice versa in the case of secular gains.

Verhaeghen employed this method on nine studies combining longitudinal and cross-sectional data on a variety of processing speed indicators. Three of the studies utilized measures of SRT. The ratio of the cross-sectional to the longitudinal slope, computed for the study of Christensen et al. (2001) is consistent with a secular decline as the ratio is <1 (0.91) indicating that the rate of longitudinal slowing is greater than the cross-sectional slowing (Figure 8.4, p. 255). The ratio computed for the study of Fozard et al. (1994) is >1 however (1.15), suggesting a lower rate of longitudinal relative to cross-sectional slowing, and therefore secular gains. For the study of Deary and Der (2005), only the choice RT ratios are presented. The SRT ratios are "censored because they were excessively large" (p. 256). Fortunately, a thorough analysis of these trends has been published elsewhere (Woodley et al., 2014b), wherein it was found that SRT has slowed across the female cohorts by -36ms over 40 years. In this analysis, a novel variant of the method proposed by Verhaeghen was developed in the form of curve-fitting, where the overall cross-sectional effect was fitted to a curvilinear function capturing the acceleration of the aging trend, then this curve was simply aligned with each pair of longitudinal observations in order to simulate their own ageing trajectories. Using the age-specific standard deviations, it was also possible to demonstrate the statistical significance of the difference between predicted and expected SRT values at different ages. In contrast, the male cohort

showed no signs of secular trends when fitted using the same curves. It was hypothesized that the younger samples (16-30) may have been strongly influenced by the relative neuro-developmental immaturity of males, who have still yet to attain peak SRT, relative to their more developmentally precocious female counterparts, which could have attenuated their longitudinal slowing trends.

The overall tendency amongst the Deary and Der (2005) data therefore leans toward a secular decline between cohorts. This can be corroborated using a more precise variant of Verhaeghen's simple linear method by computing the ratio of the decadal cross-sectional slowing trend (by taking the weighted average of all paired between-cohort differences rescaled in terms of change per decade for males and females separately), to the weighted average decadal longitudinal slowing trend within cohorts for both the male and female samples, which yields a weighted average mixed-sex ratio of 0.73, for a combined *N* of 1926 subjects. As was mentioned previously, a secular slowing trend is also evident in Christensen et al.'s (2001) data, which were collected for an *N* of 887 subjects. The Fozard et al. (1994) data (*N*=1265) are the only apparent exception, however it is predicted that once reanalysed using the curve-fitting procedure, the apparent secular gains will disappear. Interestingly, Verhaeghen (2013) reported a very large potential secular loss (ratio = 0.4) amongst Fozard et al's Go-No Go data, a measure closely related to SRT. Verhaeghen goes on to speculate, based on the pattern of results across different processing speed paradigms, that "“simpler” response time tasks show no Flynn effect, but more complex perceptual speed measures do" (p. 256). Consistent with this is the finding that the weighted average of the three SRT 'Verhaeghen ratios' is 0.9 for a combined *N* of 4078

subjects, indicating that overall, longitudinal slowing is greater than cross-sectional slowing, a finding suggestive of generational declines in SRT.

Discussion

Summary

Presented here is the strictest estimate of the prospective decline in *g* based on SRT produced to date. It tightly conforms to the analysis conducted by Dodonova and Dodonov (2013) in terms of the sorts of corrections that are applied to various studies (right down to the incorporation of the large and quite probably unjustified upwards correction to Galton's Kensington sample based on the assumption that he selected the minima of three trials). It also maximizes the comparability of the samples based on the strictest possible selection rules, i.e. each study had to have strived for high degrees of population representativeness and each study had to be large (i.e. *N*>500), so as to minimize range restriction and sampling error. Furthermore each study had to be sourced from the same country (the UK) so as to maximally reduce biocultural heterogeneity, especially with respect to cognitive differences – a known source of SRT variability between countries (see: Dutton et al., 2014 for an empirical demonstration of this). The analysis of secular trends was conducted using only known sources of between-study variance (i.e. sample size), and conversion into *g*-loss was achieved using an unadjusted standard deviation value. All of this strongly adheres to criticisms of previous analyses of ours made by Dodonova and Dodonov (2013). There is only one minor point of difference – our use of the mixed-sex samples, which is in line with Woods et al. (2015), who compare mixed sex samples

on the basis that sex differences in mean SRT are small. Relaxing this selection rule also allowed us to incorporate Wilkinson and Alison (1989) into our analysis, as they did not disaggregate their sample by sex. This was an important study to include, as it is in essence a direct replication of Galton's study, using a highly similar subject recruitment procedure.

Aggregation of 'Verhaeghen ratios' computed for three SRT studies reporting data on both longitudinal and cross-sectional performance declines, provides another independent line of evidence consistent with generational declines in SRT performance.

Conclusion

These findings enhance rather than detract from the findings of previous studies reporting secular decline trends in simple RT performance, and that as with Dodonova and Dodonov (2013), Woods et al. (2015) have helped increase the precision of our estimates of the extent of the secular trend.

Woods et al. (2015) may finally wish to consider the possibility that their SRT means overestimate of the true capabilities of the populations from which they draw their samples. Comparison of the standard deviation values obtained in their two samples (28 vs. 27ms) with those obtained in a study employing a potentially more comprehensive sample recruitment strategy (Deary et al. 2001; 119ms) makes it clear that there is considerable range restriction in the present samples. The first sample was sourced from community volunteers in Rotorua, New Zealand; the second sample

was in part recruited using advertisements in the San Francisco Bay Area Craigslist – an electronic bulletin board. These volunteers are likely to be of higher-than-average IQ, which may account for the apparent range restriction when compared with Deary et al. (2001), who sampled across a broad range of social classes. It would be interesting to compare the samples employed by Woods et al. (2015) to the mean of the populations from which they were drawn on measures like IQ, educational attainment and income.

In conclusion it is clear that Woods et al. (2015) have made an important contribution to the debate concerning the role of computer-processing lag in the inflation of contemporary estimates of SRT. They have also added to our knowledge of aging trends in cross-sectional comparisons. They have drawn together an impressive dataset, however their analyses fail to disqualify the potential reality of the secular slowing of SRT.

References

Anger, W.K., Cassitto, M.G., Liang, Y.-X., Amador, R., Hooisma, J., Chrislip, D.W., et al. (1993). Comparison of performance from three continents on the WHO-recommended Neurobehavioral Core Test Battery (NCTB). *Environmental Research, 62*, 125–147.

Cattell, J.M. (1890). Mental tests and measurements. *Mind, 15*, 373–381.

Christensen, H., Hofer, S.M., MacKinnon, A.J., Korten, A.E., Jorm, A.F., & Henderson, A.S. (2001). Age is no kinder to the better educated: Absence of an association investigated using latent growth techniques in a community sample. *Psychological Medicine, 31*, 15–28

Deary, I.J., & Der, G. (2005). Reaction time, age, and cognitive ability: Longitudinal findings from age 16 to 63 years in representative population samples. *Aging, Neuropsychology, and Cognition, 12*, 187–213.

Deary, I.J., Der, G., & Ford, G. (2001). Reaction times and intelligence differences: A population-based cohort study. *Intelligence, 29*, 389–399.

Der, G., & Deary, I.J. (2006). Age and sex differences in reaction time in adulthood: Results from the United Kingdom Health Lifestyle Survey. *Psychology and Aging, 21*, 62–73.

Dodonova, Y.A., & Dodonov, Y.S. (2013). Is there any evidence of historical slowing of reaction time? No, unless we compare apples and oranges. *Intelligence, 41*, 674–687.

Donders, F.C. (1868/1969). On the speed of mental processes. *Acta Psychologica, Attention and Performance II* (translated by W.G. Koster), *30*, 412–431.

Dutton, E., te Nijenhuis, J., & Roivainen, E. (2014). Solving the puzzle of why Finns have the highest IQ, but one of the lowest number of Nobel prizes in Europe. *Intelligence, 46*, 192-202.

Flynn, J.R. (2013). The Flynn effect and “Flynn's paradox”. *Intelligence, 41*, 851–857.

Fozard, J.L., Vercruysse, M., Reynolds, S.L., Hancock, P.A., & Quiller, R.E. (1994). Age differences and changes in reaction time: The Baltimore Longitudinal Study of Aging. Journals of Gerontology, Series B: Psychological *Sciences and Social Sciences, 49*, P179–P189

Galton, F. (1873). *Exner, Sigmund - Hirsch, A. (From Galton's notebook)*. UCL Special Collections.

Galton, F. (1885). On the anthropometric laboratory at the late International Health Exhibition. *Journal of the Anthropological Institute, 14*, 205-218.

Galton, F. (1890). Exhibition of instruments (1) for testing the perception of differences of tint, and (2) for determining reaction-time. *Journal of the Anthropological Institute, 19*, 27–29.

Garcia, J., & Quintana-Domeque, C. (2007). The evolution of adult height in Europe: a brief note. *Economics and Human Biology, 5*, 340-349.

Hatton, T.J., & Bray, B.E. (2010). Long run trends in the heights of European men, 19th-20th centuries. *Economics and Human Biology, 8*, 405-413.

Jensen, A.R. (2006). *Clocking the mind: Mental chronometry and individual differences*. Amsterdam: Elsevier.

Johnson, R.C., McClearn, G., Yuen, S., Nagosha, C.T., Abern, F.M., & Cole, R.E. (1985). Galton's data a century later. *American Psychologist, 40*, 875–892.

Lipsey, M.W., & Wilson, D. (2000). *Practical Meta-Analysis (Applied Social Research Methods)*. New York: SAGE.

Medicines for Man Organizing Committee. (1980). *Medicines for Man: A Booklet Based on an Exhibition at the Science Museum about Medicines - how They are Discovered and how They Work, how They are Made and Tested, how They are Prescribed and Dispensed, and how Laws Control Their Use.* London, Science Museum.

Nettelbeck, T.J. (2014). Smarter but slower? A comment on Woodley, te Nijenhuis and Murphy (2013). *Intelligence, 42*, 1–4.

Parker, S. (2014). Were the Victorians cleverer than us? Maybe, maybe not. *Intelligence, 47*, 1-2.

Pearson, K. (1914). *The life, letters and labours of Francis Galton*. Cambridge:

Cambridge University Press.

Silverman, I.W. (2010). Simple reaction time: It is not what it used to be. *American Journal of Psychology, 123*, 39–50.

Silverman, I.W. (2013). Testing the hypothesized effect of dysgenic fertility on intelligence with existing reaction time data: a comment on Woodley, te Nijenhuis, and Murphy (2013). *Intelligence, 41*, 664–666.

Verhaeghen, P. (2014). *The elements of cognitive aging: Meta-analyses of age-related differences in processing speed and their consequences*. Oxford: Oxford University Press.

Wilkinson, R.T., & Allison, S. (1989). Age and simple reaction time: Decade differences for 5,324 subjects. *Journal of Gerontology, 44*, 29–35.

Woodley, M.A., Madison, G., & Charlton, B.G. (2014b). Possible dysgenic trends in simple visual reaction time performance in the Scottish Twenty-07 cohort: A reanalysis of Deary & Der (2005). *Mankind Quarterly, 55*, 110-124.

Woodley, M.A., te Nijenhuis, J., & Murphy, R. (2013a). Were the Victorians cleverer than us? The decline in general intelligence estimated from a meta-analysis of the slowing of simple reaction time. *Intelligence, 41*, 843–850.

Woodley, M.A., te Nijenhuis, J., & Murphy, R. (2013b). A response to two critical

commentaries on Woodley, te Nijenhuis and Murphy (2013). *Psychological Comments* (url: http://drjamesthompson.blogspot.co.uk/2013/05/a-response-to-two-critical-commentaries.html).

Woodley, M.A., te Nijenhuis, J., & Murphy, R. (2014a). Is there a dysgenic trend towards slowing simple reaction time? Responding to a quartet of critical commentaries. *Intelligence, 46,* 131-147.

Woodley of Menie, M.A. (2015). How fragile is our intellect? Estimating losses in general intelligence due to both selection and mutation accumulation. *Personality and Individual Differences, 75*, 80-84.

Woodley of Menie, M.A., te Nijenhuis, J., & Murphy, R. (2015). Are signal luminances and confounded stimuli a source of slowing simple reaction time and cross-study heterogeneity? A response to Parker (2014). *Intelligence, 49,* 23-24.

Woods, D.L., Wyma, J.M., Yund, E.W., Herron, T.J., & Reed, B. (2015). Factors influencing the latency of simple reaction time. *Frontiers in Human Neuroscience, 9*:131.
